# Serum cholesterol levels and postoperative atrial fibrillation

**DOI:** 10.1186/1749-8090-9-69

**Published:** 2014-04-08

**Authors:** Mehmet Aydin, Ibrahim Susam, Baris Kilicaslan, Münevver Dereli, Mustafa Sacar, Oner Ozdogan

**Affiliations:** 1 Department of Cardiology, Tepecik Research and Training Hospital, Izmir, Turkey; 2 Department of Cardiovascular Surgery, Kocatepe University, Afyon, Turkey; 3 Department of Cardiovascular Surgery, 18 Mart University, Çanakkale, Turkey; 4 Manavkuyu mah. 249/4 sokak No: 2 A blok D 16 Bayraklı, İzmir, Turkiye

**Keywords:** Arterial fibrillation, Coronary artery bypass, Electrocardiography, Lipoproteins, Hypercholesterolemia

## Abstract

**Background:**

Post-operative atrial fibrillation is an important complication after coronary bypass surgery. As inflammation and oxidative stress were makedly encountered in the etiology, high cholesterol was also defined to provoke atrial fibrillation. In this present study, the relationship between postoperative atrial fibrillation and preoperative serum lipid levels were evaluated.

**Methods:**

A total of 100 patients, who were operated at the department of Cardiovascular Surgery of our hospital were included to the study analysis. Patients, who had preoperative atrial fibrillation, thyroid dysfunction, or left atrial dilatation (above 4.5 cm) were excluded from the study. Patients were divided into two groups with postoperative atrial fibrillation development (Group I n = 36), and without atrial fibrillation development (Group II n = 64). Preoperative routine blood analyses, ECG, echocardiography were evaluated. Patients were followed for atrial fibrillation development for one month starting from the intensive care unit at the postoperative period. Serum lipid profiles and thyroid function were measured. For homogenization of inflammatory factors and oxidative stress, treatments other than statins, betablockers, calcium channel blockers, aspirin, ACE inhibitors, and ARB were stopped for 10 days. Atrial fibrillation for at least ≥5 minutes in the intensive care unit was accepted as postoperative atrial fibrillation.

**Results:**

Demographic data were similiar between groups (p > 0.05). There was no difference in TC levels between groups, whereas LDL-C levels were statistically lower in patients developing post-operative atrial fibrillation (106.67 ± 28.36 vs 118.75 ± 27.75; P < 0.05).

**Conclusion:**

The more lowered is the LDL-C in the preoperative period, the more reduced risk of postoperative atrial fibrillation development. High levels of LDL-C in the preoperative period could be predictor of atrial fibrillation development in the post operative period.

## Background

Atrial fibrillation (AF) constitutes a broad area of intense daily practice for physicians in cardiac arrhytmias. Postoperative AF differ not only in its development among cases with myocardial revascularization, but also it has an incidence of 11-40%. This ratio is evaluated as a complication, which reaches up to 50% among patients with valvular surgery [1,2]. Moreover, it is the most common complication, which is encountered during the intensive care unit stay. It causes increased mechanical ventilation requirement as well as increased morbidity and mortality rates by causing vasoactive drug usage [3-5]. Because postoperative AF has some pathogenetic differences, it is considered as a model contributing in understanding AF mechanisms. In large scale studies, the reported incidence is up to 65% in cases with open cardiac surgery, and it has been reported that transient causes have been involved in the condition, because recurrence was very low in the majority of the cases. Because electroanatomic abnormalities related to atrial and/or pulmonary vein, which are basicly required for initiation and continuity of AF are not permenant in these cases, the recurrence is quite low [6]. Marked predictors of postoperative AF can be named as age, low ejection fraction, intraoperative technical reasons, and inflammatory mechanisms [4,7,8]. Along with the direct relationship between serum cholesterol levels and atherosclerotic plaques, its correlation has been found particularly with ventricular arrythmias and atrial fibrillation [9,10]. Changes in cholesterol content of cellular membrane may cause formation of the substrate, which can initiate atrial fibrillation by changing cellular membrane characteristics, and obtained evidences have supported that this may be caused by cellular membrane specific ion channels and pump functions acting the efficacy of hormone specific receptors. When ion channel characteristics have differed, Ca^2^ accumulation in the cellular membrane and sudden changes in the effective refractory period may cause initiation of AF [11-13]. Therefore, electrical remodelling, which is considered necessary for continuity of AF, is formed. Serum preoperative LDL-C levels could be beneficial in predicting for postoperative AF independently from lipid lowering treatments. Although it is well known that preoperative intense statin treatments could prevent postoperative AF development, detection of low serum LDL-C level effect on cardiac myocytes could also be an important finding. Based on these findings, we investigated the correlation between postoperative AF and serum lipid levels in our study.

## Methods

A total of 100 cases, which had bypass operation at our Cardiovascular Surgery clinic, were included in this prospective study. The exclusion criteria for cases were presence of preoperative AF, left atrial dilation above 4.5 cm, valvular heart disease, thyroid dysfunction, emergency bypass requirement, prior surgical maze and pace-makers. Patients, who required valvular replacement at the same session, were included in the study if the left atrial dilation was not above 4.5 cm. Fasting blood samples of 12 hours were collected during preoperative preparation period from all participants to perform routine blood analyses and serum lipid level measurement. Concomitant beta-blocker and dyslipidemia treatment was continued in pre- and postoperative periods. ECG and electrocardiographic evaluations were performed in all participants (left atrium period, left ventricular width and ejection fraction were investigated). Variables related to surgical procedure were defined and recorded (pump duration, number of vessels underwent bypass surgery). After the bypass surgery, cases followed up for at least 48 hours in the intensive care unit had ECG recordings, and if AF was developed, then they were defined and recorded. Meanwhile, although ECG recodings were performed at 12 hours intervals, if there was any complaint, the ECG was recorded.

Blood samples of cases were collected in the operation morning after 12-hour fasting to examine serum lipid profile, thyroid function tests, and fasting blood glucose levels. ECG recording of each case was performed, and preoperative sinus rhythm was recorded. Serum lipid levels were measured by using Hitachi modular D_P analyzer (Manhaim/Germany).

Our study was approved by the local ethics committee of Pamukkale University School of Medicine. Our research is in compliance with the Helsinki Declaration.

## Statistical methods

Statistical analysis was performed using SPSS 11.0 for Windows. Data are presented as mean ± SD, controlled for normally distribution by Kolmogorov-Smirnov test. Comparison of more than two groups was performed by using Kruskal-Wallis test with Bonferroni correction and differences between any two groups were compaired by Mann-Whitney *U* test because of abnormal distribution. Categorical data between two or more groups were compared by the Pearson χ2 test. A probability value of p < 0.05 was considered as significant.

## Results

Cases underwent bypass surgery (n = 100) were divided as Group I (n = 36, 36%) (cases developed AF postoperatively) and Group 2 (n = 64, 64%) (cases without AF postoperatively). 36% of patients were developed AF after the bypass surgery. There was no statistically significant difference in demographic data except age between the groups (P < 0.05). We believe that marked homogeneity has been provided between two groups, because there was no statistically significant difference in statin usage between the groups. Groups and statistically analyzed demographic data are given in Table 1. There was no statistically significant difference in biochemical results between groups except LDL cholesterol (LDL-C). (118.75 ± 27.75 vs 106.67 ± 28.36; P < 0.05) (Table 2).

**Table 1:** Demographic characteristics of patients

	**Group I**	**Group II**
	**(n = 36)**	**(n = 64)**
Age*	68.50 ± 8.05	62.40 ± 10.52
Gender (female/male)	10/26	13/51
Family history	6 (16.7%)	15 (23.4%)
Smoking history (10 years, 5 cigarettes/d)	20 (55.6%)	32 (50.0%)
Hypertension (BP >139/89 mmHg)	20 (55.6%)	33 (51.6%)
Diabetes mellitus (DM)	11 (30.6%)	25 (39.1%)
Carotis artery lesion	4 (11.1%)	3 (4.7%)
Periferal artery disease	5 (13.9%)	6 (9.4%)
Angina pectoris	20 (55.6%)	41 (64.1%)
Myocardial infarction	5 (13.9%)	19 (29.7%)
Beta-blocker use	19 (52.8%)	34 (53.1%)
Statin use	7 (19.0%)	13 (20.3%)
COPD (using steroid)	5 (13.9%)	4 (6.2%)
Left ventricular ejection fraction (%)	51.63 ± 12.43	50.28 ± 9.88
Body mass index	25.41 ± 3.47	26.64 ± 3.76

*P value for Age is significant (< 0.05) whereas the values are insignificant for the rest.

**Table 2:** Comparison of preoperative lipid profiles and biochemical examinations between the groups

	**Group I**	**Group II**
	**(n = 36)**	**(n = 64)**
WBC	9.27 ± 3.26	9.20 ± 3.32
Hemoglobin	13.22 ± 1.79	13.12 ± 1.87
Hematocrit	38.98 ± 5.39	38.72 ± 5.72
Platellet	252.88 ± 98.16	241.75 ± 80.48
Creatinine, mg/dl	1.08 ± 0.96	1.37 ± 1.63
BUN, mg/dl	20.35 ± 10.98	21.49 ± 17.21
Glucose, mg/dl	129.05 ± 55.96	152.20 ± 68.06
AST, mg/dl*	24.23 ± 14.73	40.65 ± 54.97
ALT, mg/dl	30.51 ± 62.58	33.54 ± 42.46
GGT, U/L	29.77 ± 31.83	39.23 ± 22.57
LDH, mg/dl	275.72 ± 107.32	291.51 ± 167.61
CRP, mg/dl	2.50 ± 3.34	1.67 ± 2.38
Total cholesterol, mg/dl	178.72 ± 46.37	178.27 ± 42.95
LDL-C, mg/dl*	118.75 ± 27.75	106.67 ± 28.36
HDL-C, mg/dl	36.11 ± 9.09	35.80 ± 10.62
Triglyceride, mg/dl	147.17 ± 69.05	183.58 ± 114.11
VLDL-C, mg/dl	28.02 ± 10.90	44.51 ± 66.00

*P value for Age is significant (< 0.05) whereas the values are insignificant for the rest.

Along with significant difference in preoperative LDL-C values between the two groups, marked difference in triglyceride (Group I = 147.17 ± 69.05 mg/dl, Group II = 183.58 ± 114.11 mg/dl; p > 0.05) and VLDL cholesterol (VLDL-C) arm (Group I = 28.02 ± 10.90 mg/dl, Group II = 44.51 ± 66.00 mg/dl; p > 0.05) was noteworthy despite the statistical insignificance.

## Discussion

AF is a continuous cardiac arrhythmia affecting 0.9% of the population, and studies conducted to enlighten the underlying mechanisms have also warranted innovations in the treatment. In the development of AF, complex relationships are considered among arrhythmogenic substrate, triggering factor(s), autonomic nervous system [14]. Oxidative stress and inflammation have a significant role in AF development have been evolved [15]. Bruin et al. enforced the inflammation hypothesis by showing the relationship between postoperative AF and C-reactive protein (CRP) level for the first time [16]. Marked increase in CRP level in persistent AF when compared with paroxysmal AF suggests that inflammation has a more significant role in determining the continuity in AF process rather than a triggering role [17]. In this situation, opinion of the triggering factor presence stands in the foreground in postoperative AF. It may be considered that this hypothesis has been supported by the absence of correlation between preoperative CRP levels and postoperative AF in our study.

Role of oxidative stress in pathogenesis of AF has been shown in human and animal models. Signs related to electrical remodeling and oxidative damage have been rather at the experimental level. Particularly vitamin C; an antioxidant substance, was reported to prevent AF in preoperative cases both in human and animal models [18].

Postoperative AF occurs usually on postoperative 2-3 days [16]. Intense researches have been performed for its prevention, because it has increased the unfavorable results such as stroke, congestive heart failure, elongation of hospital stay, and increased need for vasoactive agents. Majority of these studies have mainly focused on inflammation suppression and agents decreasing the oxidative stress. Dotani et al. showed that postoperative AF incidence was markedly decreased by preoperative load of statins [19]. Ozaydınlı et al. proved that duration of postoperative AF was also decreased by statin usage [20]. When all statin studies were evaluated, we noted that the relationship between preoperative LDL-C levels and postoperative AF frequency was not sufficiently investigated. Marked increase in LDL-C levels in postoperative AF cases when compared to the ones without AF has indicated that causative relationship should be investigated.

Cellular membrane and cholesterol content form a dynamic structure. Changes in membrane cholesterol content can change functional properties of the membrane. Although formation of these changes has not been totally understood yet, three mechanisms have been proposed: 1) relationship between cholesterol and membrane proteins, 2) modulation of membrane fluidity, and 3) relationship between lipid islets and proteins in the membrane [21]. These properties of membrane can be controlled by decreasing serum cholesterol content, and excitability can be decreased. There are a few studies in the literature reporting such effects of statins on membrane proteins. It has been suggested that antiarrhythmic effect has been exerted through intracellular calcium homeostasis by various statin structures at various levels, because intracellular mevalonate-dependent cholesterol synthesis was inhibited by statins [22]. We believe that low levels of serum cholesterol slow down intracellular LDL-C intake, and also slow down free cholesterol level through lysosomes. Because membrane lipid content equilibrium is saved, formation of arrhythmic potentials may be prevented by the above mentioned mechanisms. Low serum LDL-C levels, providing the cellular membrane stability, can inhibit electrical remodeling by affecting intracellular Ca levels of triggering factors. It was reported that oxidation products were eliminated and endothelial functions were recovered in hypercholesterolemic cases by applying LDL apheresis once [23,24]. When pleotropic effects of statins have been excluded, it may be remarked that determination of LDL-C in postoperative cases; decrease of LDL-C by apheresis application, if the levels are high; and having the operation are the preventive factors in AF development.

The patients in group 1 were older than the patients in group 2. This is the main limitation of our study. Because this could affect AF prevalence in group 1. However, there was no statistically significant difference in other demographic data and we believe that marked homogeneity has been provided between these two groups.

## Conclusion

In conclusion, we think that serum preoperative LDL-C levels can be beneficial in predicting for postoperative AF independently from whether patients have received any lipid lowering treatments. Although it is known that preoperative intense statin treatments can prevent postoperative AF development, we believe that detection of low serum LDL-C level effect on cardiac myocytes is an important finding. We believe that further large scale studies are required to evaluate this argument.

## Competing interests

All authors declare that there is no conflict of interest in the article.
